# Managing Aerodigestive Emergencies During the COVID-19 Pandemic: Challenges for Healthcare Workers

**DOI:** 10.21315/mjms2020.27.3.17

**Published:** 2020-06-30

**Authors:** Madhusudhan Krishnamoorthy, Mohd Shaiful Nizam Mamat Nasir, Irfan Mohamad

**Affiliations:** Department of Otorhinolaryngology-Head & Neck Surgery, School of Medical Sciences, Universiti Sains Malaysia, Kubang Kerian, Kelantan, Malaysia

Dear Editor,

We read with great interest the special editorial entitled ‘A critical appraisal of COVID-19 in Malaysia and beyond’ that was recently published in the *Malaysian Journal of Medical Sciences* (1). The article covers almost all aspects of the coronavirus disease 2019 (COVID-19) pandemic scenario from the Malaysian perspective. Based on our PubMed search, the article is among the first to consider COVID-19 in Malaysia. We wish to express our congratulations and to thank you for such a valuable contribution to science.

The subheading ‘Challenges to healthcare workers’ really captured our attention, particularly in terms of paragraph six of the article, in which it is stated that the issue of surgical emergencies during the pandemic requires an immediate consensus and the development of guidelines, especially for those who require early surgical treatment (1). In light of this, we would like to share a case that caught our attention in the middle of the pandemic (on day 16 following the implementation of the 2020 Movement Control Order [MCO]).

A 35-year-old female patient presented at the emergency department complaining of having ingested a fish bone some 5 h prior. She was eating fried croaker fish with rice when the ingestion occurred. The patient claimed that she was unable to detect the fish bone in her mouth due to incomplete chewing as a result of a concomitant but unrelated traumatic ulcer on the inner surface of her lower lip. Post-ingestion, she complained of a persistent, sharp pricking pain in the throat, which was aggravated each time she swallowed her saliva. The pain did not radiate elsewhere. Otherwise, there was no change in the patient’s voice, no noisy breathing and no swelling overlying the neck. She had not attempted to remove the fish bone. The patient was able to use her finger to pinpoint the exact location of the pain as the midline of the anterior neck slightly above her suprasternal notch. Aside from having undergone axillary clearance under general anaesthesia (GA) for accessory breast tissue a few years earlier, the patient’s prior medical history was not significant.

Upon examination, the patient was alert, comfortable, afebrile and in no distress. There was no hoarseness and no stridor, and her vital signs were stable. Laryngeal crepitus was present, and there was no neck swelling or local temperature increase following palpation. A radiograph of the soft tissue of her neck (lateral view) revealed a thin and linear radiopaque foreign body within the prevertebral soft tissue between the C6 and C7 vertebra. An air oesophagogram was performed extending from the lower level of the cervical 5 vertebra to the lower level of the cervical 7 vertebrae (Figure 1). Otherwise, the prevertebral soft tissue space was not widened, and there was no loss of cervical lordosis.

In line with the Ear, Nose and Throat (ENT) UK’s COVID-19 - Adult Nasoendoscopy (FNE) and Possible Upper Aerodigestive Tract Fish Bone Investigation protocol (2), ‘emergent FNE by the senior decision maker’ was not performed because the likely ingested fish bone had passed below the level of the cricopharyngeal sphincter. Thus, there was no clear indication for the routine endoscopic ENT assessment should be performed in the clinic or emergency department (3). Another challenge faced by the team concerned the decision to screen for the COVID-19 virus by taking a nasopharyngeal swab. At this point in time (day 16 following the implementation of the 2020 MCO), there were no local guidelines regarding the need to swab patients undergoing urgent surgery under GA. Therefore, based on the targeted and appropriate history taking, the patient was admitted after all the risk factors had been ruled out. The decision to not perform a screening test was made on the basis of avoiding wasting COVID-19 screening kits and related resources. The patient underwent direct laryngoscopy and oesophagoscopy under GA on the same day.

Intraoperatively, a sharp and pointed fish bone was found to be embedded in the posterior wall of the patient’s oesophagus, some 18 cm from her upper central incisor (Figure 2). There were only minimal mucosal abrasions and bleeding, which were secured by packing with adrenaline patties. Otherwise, no other injury or ulcer was seen within the patient’s oesophagus after the rigid oesophagoscope was advanced up to 23 cm or upon the withdrawal of the oesophagoscope. The post-operative period was uneventful, and the patient was discharged the following day.

Special precautionary measures were taken to ensure the safety of the entire surgical team as well as of the patient. These measures included the wearing of an N95 mask, the use of a face shield and the donning of a proper operating-theatre gown throughout the surgical procedure (4). After the procedure, all members of the team washed their hands with soap and water for at least 20 s, as recommended by the Centers for Disease Control and Prevention (CDC).

A risk assessment represents a very important step in relation to the judicious use of personal protective equipment (PPE). In addition to intubation being a known aerosol-generating procedure (AGP) associated with an increased risk of the transmission of respiratory infection (5), the upper gastrointestinal endoscopy procedure, including laryngoscopy and oesophagoscopy, is plausibly considered to be an AGP despite the lack of relevant evidence (6). When managing those patients with either confirmed or suspected COVID-19 status, surgical teams who are performing these procedures need to wear a filtering facepiece (FFP) 2 respirator mask, eye protection, gloves and a long-sleeved gown.

## Figures and Tables

**Figure 1:**
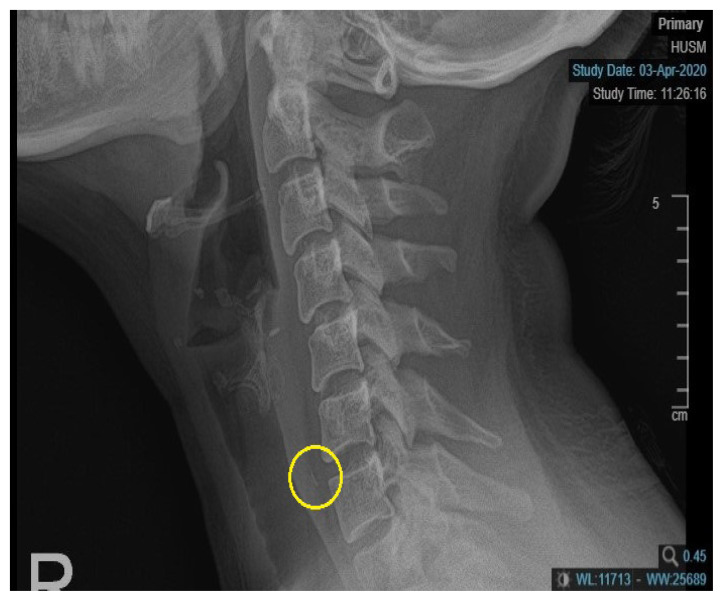
Opaque foreign body seen at the level C6–C7 with oesophagogram

**Figure 2:**
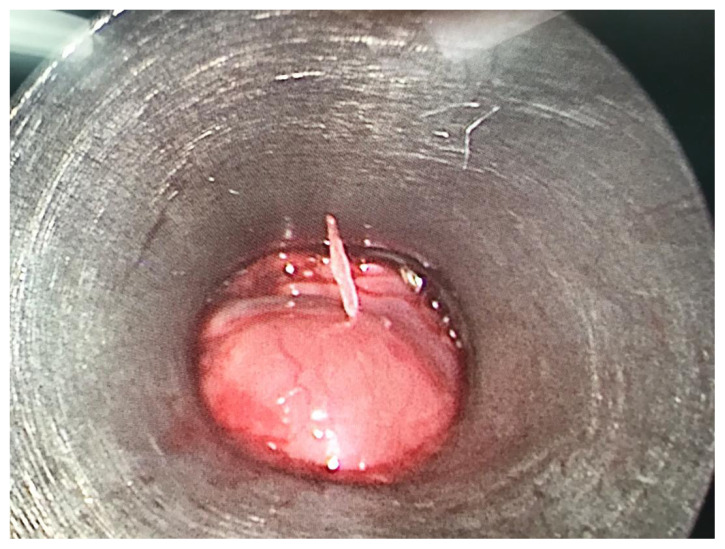
Fish bone piercing the posterior oesophageal wall as seen from oesophagoscope
